# Future Frontiers in Bioinspired Implanted Biomaterials

**DOI:** 10.1002/adma.202506323

**Published:** 2025-07-30

**Authors:** Qi Gu, Rui Yuan, Dadi Sun, Gordon Wallace

**Affiliations:** ^1^ Human Organ Physiopathology Emulation System State Key Laboratory of Organ Regeneration and Reconstruction Institute of Zoology Chinese Academy of Sciences Chaoyang District Beijing 100101 P. R. China; ^2^ Beijing Institute for Stem Cell and Regenerative Medicine Chaoyang District Beijing 100101 P. R. China; ^3^ Intelligent Polymer Research Institute Innovation Campus University of Wollongong Squires Way North Wollongong NSW 2500 Australia; ^4^ University of Chinese Academy of Sciences Huairou District Beijing 101408 P. R. China; ^5^ Beijing TongRen Hospital of Capital Medical University Dongcheng District Beijing 100051 P. R. China

**Keywords:** bioinspired materials, implantation, tissue engineering

## Abstract

Bioinspired materials draw design inspiration from nature's principles and integrate them with engineering requirements to construct highly functional and complex systems across multiple length scales. Bioinspired implanted biomaterials are highly promising in regenerative medicine, being designed to integrate customized materials with biological functions to replicate the complexity of living tissues. Organs are dynamic, multi‐interface architectures with intricate mechanical, biochemical, and physiological properties, posing a major challenge for accurate replication. This perspective explores recent advancements in the design of natural and synthetic biomaterials, focusing on strategies like cell‐laden scaffolds and cell‐free constructs, which interact dynamically with the body's microenvironments to promote tissue regeneration. How smart biomaterials that respond to biological stimuli are reshaping material functionalization, offering long‐term therapeutic solutions is examined. Additionally, how innovations in 3D printing, nanotechnology, and personalized medicine are **overcoming current barriers and improving clinical use**. Overcoming the challenges associated with replicating complex tissue structures, along with technological advancements, will be crucial to unlocking the full clinical potential of bioinspired implanted biomaterials.

## Graphical abstract

Gu et al. present an integrative overview of cutting‐edge strategies in bioinspired implantable biomaterials for organ regeneration, highlighting how emerging approaches—including 3D bioprinting, scaffold design, hydrogel systems, surface modification, nanofiber engineering, and genetic manipulation—converge to restore structure and function across diverse tissues.

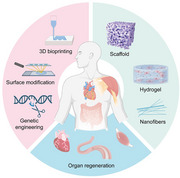

## 1 Introduction

**Figure 1:**
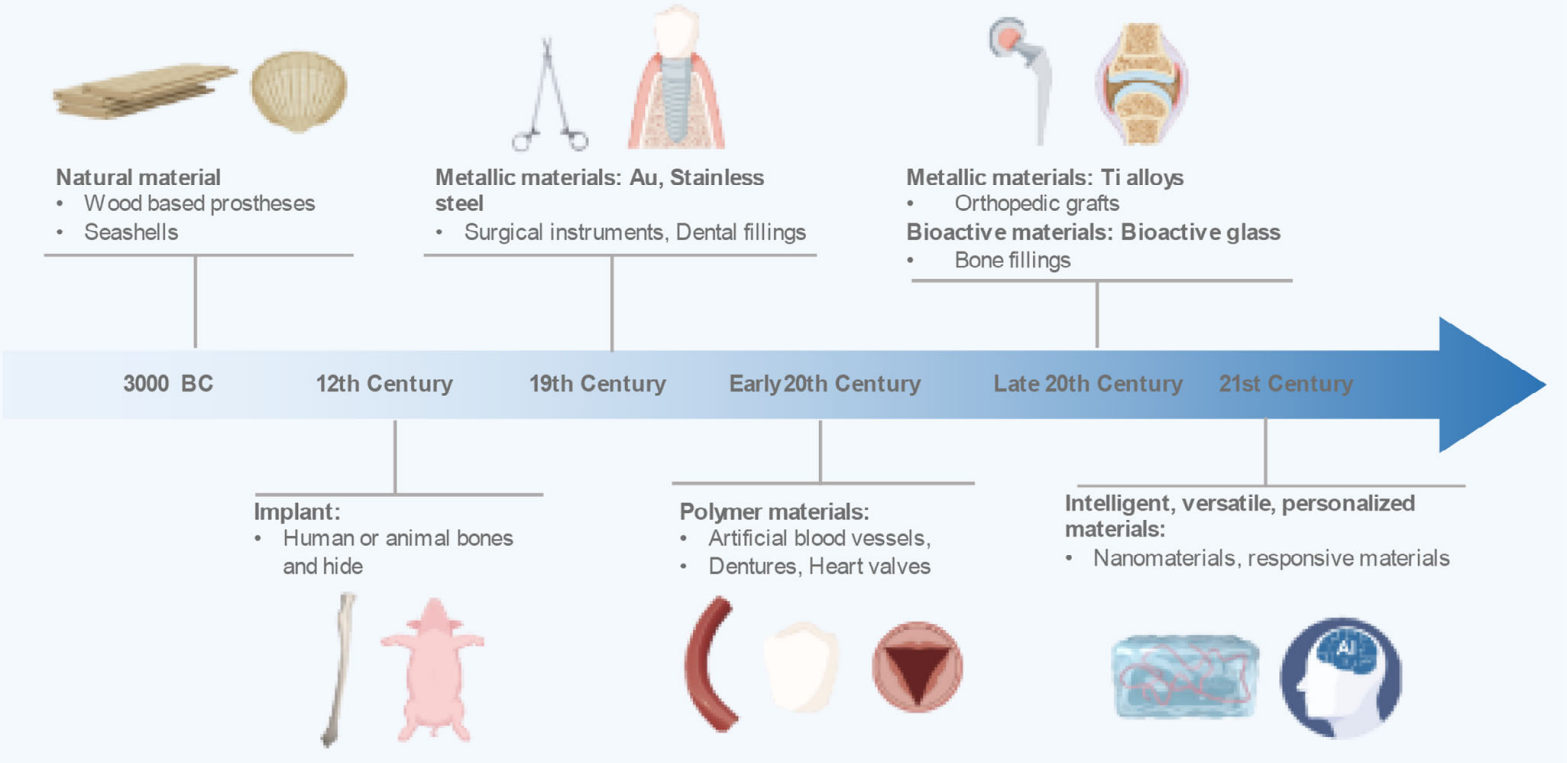
Evolutionary timeline of implantable biomaterials depicting advances in biomaterials from 3000 BC wooden prostheses to the 21st Century bio‐hybrid and responsive materials. Partial graphical elements were adapted from HOME for Researchers (https://www.home‐for‐researchers.com/#/).

Development of materials for medical applications spans thousands of years, reflecting humanity's continuous drive for innovations to repair and enhance biological functions (**Figure** **1**). The earliest records of bone repair occur in ancient Egypt around 3000 BC, when people began using simple implants such as wooden prostheses to replace missing limbs.^[^ ^1^ ^]^ Over the centuries, the scope of implant materials expanded as civilizations advanced their understanding of materials science and medicine. During the medieval period, there were early surgical attempts at bone repair, including using pieces of human or animal bones to replace damaged bone tissue, as seen in historical reports dating back to the 12th century.^[^ ^2^ ^]^ Though their success was limited due to a lack of advanced techniques and sterile practices, these early efforts in bone repair were vital in establishing a foundation to develop modern methods of tissue regeneration. By the 20th century, breakthroughs in metallurgy and polymer science enabled the development of modern implantable biomaterials.^[^ ^3^ ^]^ Titanium and its alloys (e.g., Ti6Al4V) have been used since the 1950s due to their excellent mechanical properties and biocompatibility, becoming the standard for orthopedic and dental implants.^[^ ^4^ ^]^ Recent advances in metallic biomaterials focus on mechanothermal (e.g., sandblasting, grinding, plasma spraying, laser treatment) and physicochemical (e.g., acid etching, anodization, bioactive coatings like hydroxyapatite, peptides, and antimicrobials) modifications to improve biocompatibility, corrosion resistance, antimicrobial efficacy, and osteointegration.^[^ ^5^ ^]^ These surface engineering innovations are driving the development of next‐generation biomaterials with enhanced functionality and durability.^[^ ^5^, ^6^ ^]^

Alongside metallic biomaterials, the development of synthetic polymers, such as polymethyl methacrylate (PMMA) for bone cement and polyethylene for joint replacements, is advancing cell‐free scaffolds development and **broadening clinical use** of implantable biomaterials.^[^ ^7^ ^]^ Such polymers offer mechanical strength, durability, and stability, playing a critical role in orthopedic, dental, and soft tissue applications.^[^ ^7b^ ^]^ Furthermore, nanomaterials have been widely investigated in tissue engineering, medical implants, and biosensing. The specific chemical bonds in nanocomposites provide mechanical strength and other properties to the biological materials, making them more stable and capable of sustained drug delivery.^[^ ^8^ ^]^ For instance, ultra‐high molecular weight polymers and their nanocomposites (such as functionalized single‐walled carbon nanotubes, f‐SWCNTs) have also been widely used in artificial implants for knee and hip joints due to their excellent mechanical properties, fatigue resistance, and fracture toughness in recent years.^[^ ^9^ ^]^ To enhance biological functionality, bioactive polymer composites and degradable scaffolds have been developed to modulate host cell behavior.^[^ ^10^ ^]^ Among these, hydrogel‐based scaffolds have been developed for controlled drug release, including enzyme‐responsive hydrogels that degrade in response to matrix metalloproteinases (MMPs), enabling precise, localized drug delivery for wound healing and cancer therapy.^[^ ^11^ ^]^ Chemically responsive hydrogels, such as glucose‐sensitive hydrogels, adjust insulin release in response to blood glucose fluctuations, **providing a regulated approach to diabetes control**.^[^ ^12^ ^]^

While cell‐free scaffolds deliver key mechanical and biochemical signals for tissue integration, their dependence on host cell infiltration and remodeling limits effectiveness, particularly in poorly vascularized or slow‐healing environments.^[^ ^13^ ^]^ This challenge has driven the parallel development of cell‐laden biomaterials to actively support tissue regeneration by incorporating living cells, enabling a more dynamic integration with the host tissue.^[^ ^14^ ^]^ By embedding living cells within biomimetic matrices, these bioengineered constructs not only replicate the extracellular matrix (ECM) but also provide mechanical support and biochemical cues that promote cell adhesion, differentiation, and vascularization.^[^ ^15^ ^]^ Recent innovations include bioceramic‐polymer scaffolds for bone regeneration,^[^ ^16^ ^]^ hydrogel‐based bioinks for 3D bioprinting complicated constructs,^[^ ^17^ ^]^ and bioengineered blood vessels for vascular grafting,^[^ ^18^ ^]^ some of which are already in clinical use. The progression of both cell‐free and cell‐laden biomaterials marks a paradigm shift toward bioactive, regenerative implants.

As the field of implantable biomaterials continues to advance, there is **a growing need for materials that dynamically** interface with biological environments to monitor, adapt, and respond to real‐time physiological changes.^[^ ^19^ ^]^ Smart biosensing devices, which are equipped with embedded sensors, could track biochemical and mechanical signals, including fluctuations in pH, inflammatory markers, and mechanical stress, allowing for timely therapeutic modulation. The integration of stimuli‐responsive materials, such as electroactive polymers (e.g., Poly(3,4‐ethylenedioxythiophene), PEDOT),^[^ ^20^ ^]^ piezoelectric nanomaterials,^[^ ^21^ ^]^ and magnetically responsive scaffolds,^[^ ^22^ ^]^ facilitates applications beyond chronic disease management, enabling extension to neural stimulation, dynamic wound healing, and adaptive orthopedic implants.^[^ ^23^ ^]^ These materials are also driving progress in chronic disease management, post‐surgical recovery, and regenerative medicine.^[^ ^24^ ^]^

Currently, implantable biomaterials still encounter challenges in reaching long‐term functionality, biological integration, and adaptability across diverse patient populations despite the rapid development.^[^ ^25^ ^]^ While structural scaffolds and cell‐laden biomaterials have enhanced tissue regeneration and functional restoration, challenges like immune rejection, fibrosis, limited vascularization, and mechanical mismatches persist, impeding clinical success.^[^ ^26^ ^]^ The transition toward smart biomaterials capable of real‐time monitoring and adaptive responses introduces complexities in material design, biocompatibility, and device integration.^[^ ^27^ ^]^ In particular, the transition reflects a broader conceptual shift toward bioinspiration, where material design is guided by the abstraction of functional principles from natural systems. Unlike biomimicry, which attempts to directly replicate biological forms or processes, bioinspired strategies reinterpret biological mechanisms to create innovative, adaptable solutions. Meanwhile, biohybrid materials constitute a distinct category, combining living cells with synthetic scaffolds to form interactive systems at the interface of biology and engineering. These distinctions lay the theoretical groundwork for emerging biomaterials that actively communicate with the host environment. Establishing effective communication between biomaterials and host tissue requires the development of multifunctional scaffolds that provide mechanical support, bioactivity, and sensing capabilities, all while ensuring scalability and regulatory compliance.^[^ ^28^ ^]^ The next section examines key developments in bioinspired biomaterials, highlighting the importance of cell‐laden and acellular scaffolds as well as both organ‐specific strategies in tissue engineering and regenerative medicine.

## 2 Recent Advances in Biomaterial Design: Materials for Complex Human Organs

**Figure 2:**
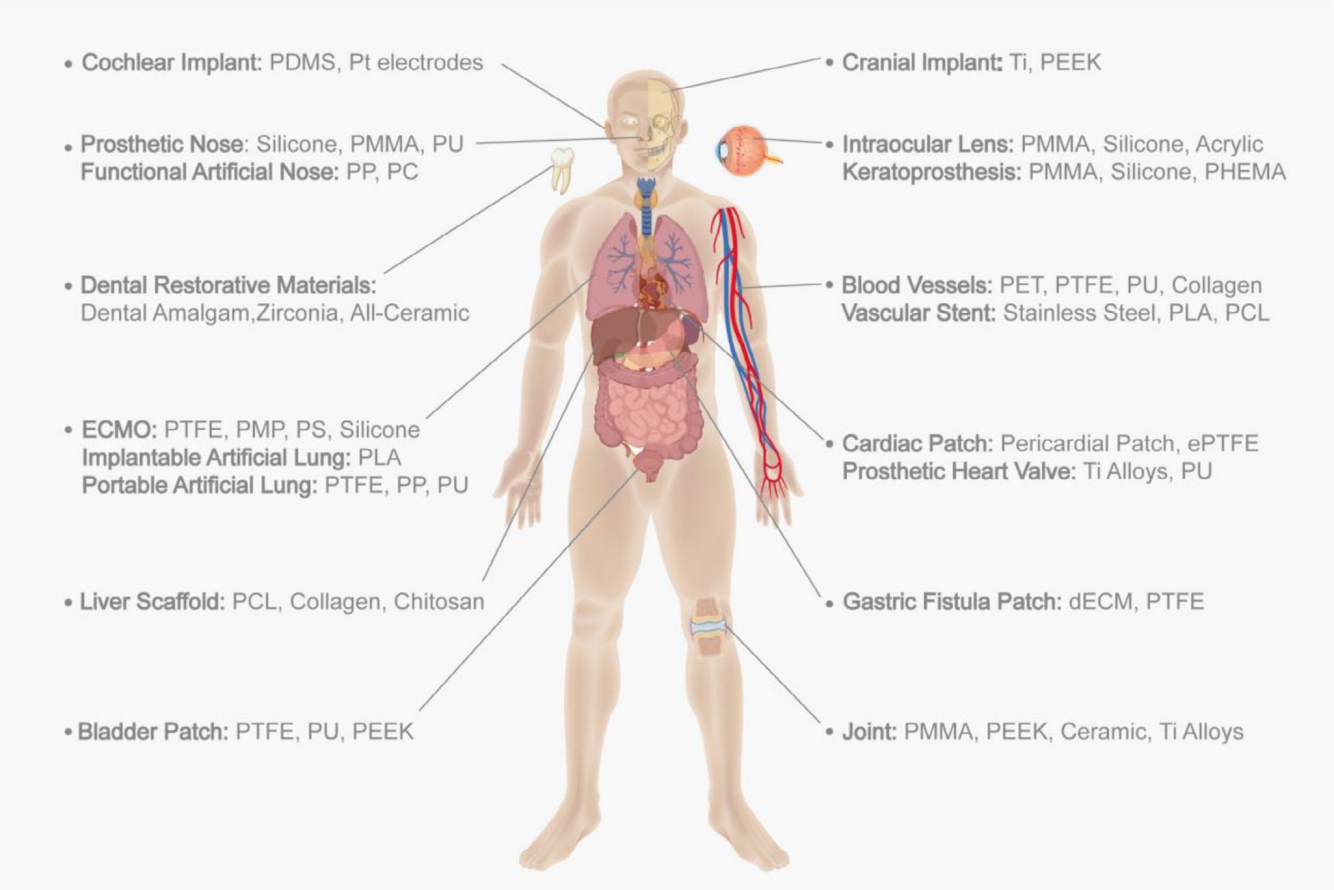
Biomaterials are used in research and in the human body parts. This figure provides a comparative anatomical overview of representative biomaterials across multiple organs and tissue systems, highlighting material‐tissue correspondence. Partial graphical elements were adapted from BioRender (https://biorender.com/).

Recent advances in biomaterial design increasingly focus on developing bioinspired materials that closely replicate the structure and function of human organs.^[^ ^29^ ^]^ As shown in **Figure** **2**, skull reconstruction presents the challenge of developing materials that simultaneously provide mechanical strength and flexibility to match the biomechanical properties of bone. Polyetheretherketone (PEEK) and titanium alloys are widely utilized in skull reconstruction due to the inherent mechanical strength and osteointegration capabilities.^[^ ^30^ ^]^ In skin regeneration, bioactive materials like collagen, hyaluronic acid (HA), and silicone‐based hydrogels promote angiogenesis, enhance wound healing, and support neovascularization.^[^ ^31^ ^]^ Clinically available skin substitutes, such as Integra and Apligraf, contain collagen and bioactive components to facilitate wound healing and tissue regeneration.^[^ ^32^ ^]^ Traditional corneal repair relies on PMMA and collagen to preserve optical transparency, while emerging approaches integrate synthetic corneal structures to enhance regenerative potential.^[^ ^33^ ^]^ Intraocular lens implants are commonly made from PMMA, silicone, and acrylates, offering long‐term optical stability and biocompatibility.^[^ ^34^ ^]^ In addition, titanium alloys are currently the first choice for dental implants due to their high mechanical strength and excellent bone integration.^[^ ^35^ ^]^ Nanocomposite material, ultra‐high‐molecular‐weight polyethylene (UHMWPE),^[^ ^36^ ^]^ has been widely studied in artificial implants for the hip and knee joints due to its excellent biocompatibility and mechanical performance, demonstrating excellent wear resistance, fatigue strength, and long‐term biocompatibility^[^ ^37^ ^]^ that make it a material of choice for load‐bearing applications such as knee and hip replacements.^[^ ^38^ ^]^

Artificial blood vessels are commonly constructed from robust yet flexible polymers like polyethylene terephthalate (PET), expanded polytetrafluoroethylene (ePTFE), and polyurethane (PU).^[^ ^39^ ^]^ For small‐diameter vessels, degradable materials such as polycaprolactone (PCL) and polylactic acid (PLA) are applied to mitigate risks such as thrombosis.^[^ ^18^, ^40^ ^]^ Rhinoplasty often utilizes materials like silicone, Gore‐Tex, and collagen for nasal dorsum augmentation.^[^ ^41^ ^]^ Hydroxyapatite, bioactive glasses, and calcium phosphate‐based materials are widely used in bone repair to enhance osteointegration and support regeneration, with biological factors like rhBMP‐2, rhBMP‐7, and platelet‐rich plasma enhancing graft effectiveness.^[^ ^42^ ^]^ For cartilage repair, scaffold‐based and scaffold‐free biomaterials, along with immunomodulatory strategies, are being developed to improve mechanical strength and withstand the inflammatory environment in osteoarthritic joints.^[^ ^43^ ^]^ Artificial heart valves are constructed from decellularized animal tissue (biological valves) or non‐biological biomaterials (mechanical valves).^[^ ^44^, ^45^ ^]^ Myocardial repair employs diverse bioactive materials, including biocompatible scaffolds and growth factor‐loaded hydrogels, to promote tissue regeneration, restore heart function, and improve mechanical properties in damaged cardiac tissue.^[^ ^46^ ^]^ Liver tissue engineering involves the use of hydrogels, both natural and synthetic, to support hepatocyte growth and function.^[^ ^47^ ^]^ Artificial lung systems, such as extracorporeal membrane oxygenation (ECMO), are designed to support gas exchange in patients with respiratory failure. Ongoing advancements aim to enhance compatibility, performance, and longevity for potential implantable applications.^[^ ^48^ ^]^

Implantable biomaterials are generally classified into metals (e.g., titanium alloys, stainless steel), synthetic polymers (e.g., PLA, PEEK, PU), and naturally sourced materials (e.g., collagen, chitosan, hyaluronic acid).^[^ ^49^ ^]^ Each class offers distinct advantages tailored to specific tissue needs: metals provide high strength and durability, ideal for bone and dental implants; polymers enable tunable degradation and are commonly used in neural and cardiovascular systems; natural biomaterials ensure superior biocompatibility and ECM mimicry, especially beneficial for skin, vascular, and cartilage repair.^[^ ^50^ ^]^

These bioinspired materials contribute to regenerative medicine by replicating native tissue properties, enabling effective organ repair and regeneration. Implantable constructs now integrate biomaterials designed for specific tissues, supporting regeneration through cell‐laden scaffolds, which incorporate living cells for active repair, or cell‐free scaffolds, which provide structural support and promote host cell migration.

## 3 Implanted Constructs and the Distinction between Cell‐Laden and Cell‐Free Approaches

**Figure 3:**
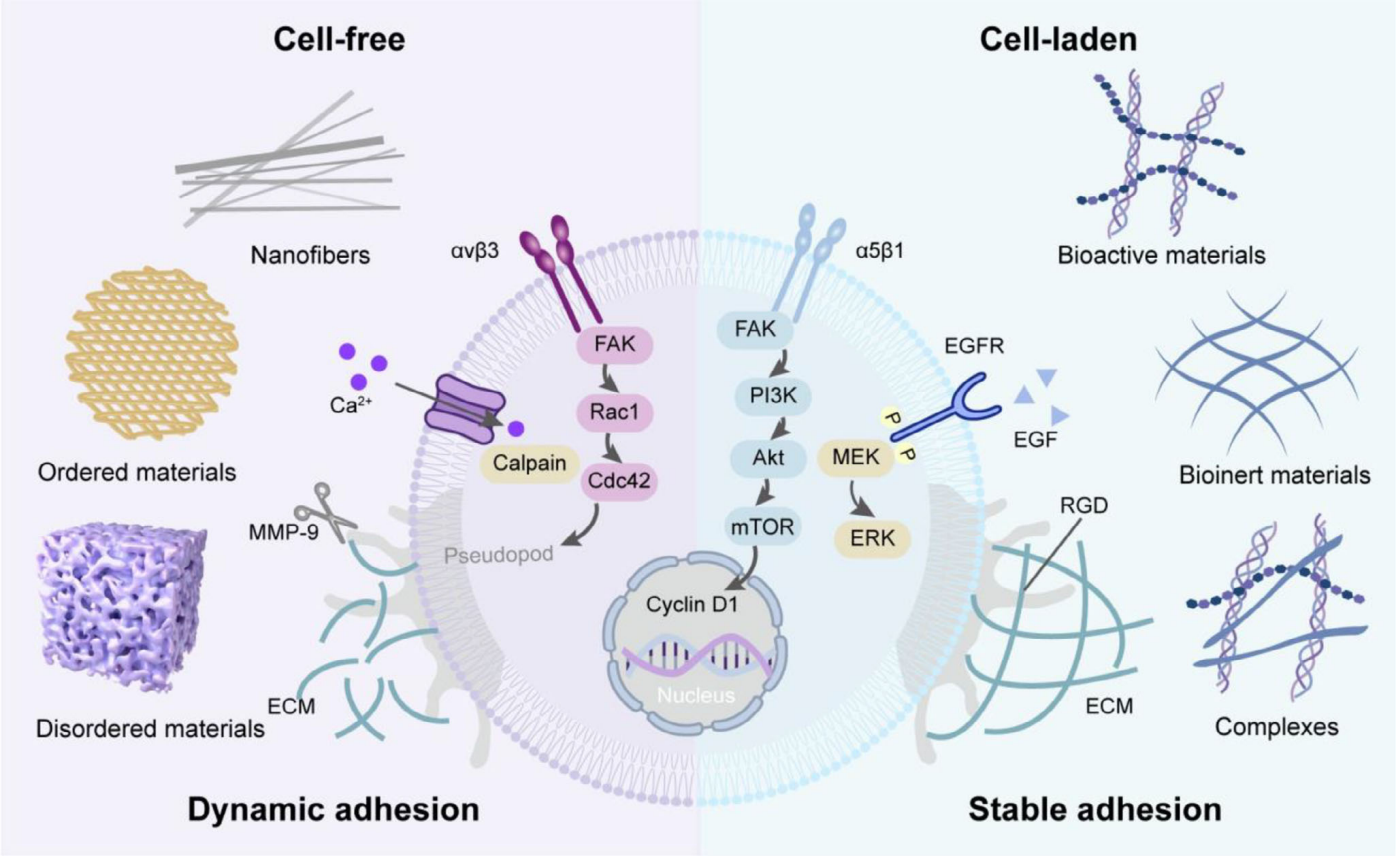
The distinctions between cell‐free and cell‐laden transplantation strategies. Partial graphical elements were adapted from HOME for Researchers (https://www.home‐for‐researchers.com/).

Each adult organ is composed of billions of cells, which are the fundamental building blocks, organized into highly complex, tissue‐specific architectures that perform essential physiological functions. Implanted scaffolds provide physical support and guide the growth of cells in tissue engineering. The integration of living cells into scaffolds, as well as the use of acellular scaffolds that facilitate natural tissue regeneration, offers unique advantages depending on the target organ and application.^[^ ^51^ ^]^ Cell‐laden and cell‐free scaffolds are essential in tissue engineering, with their roles and effectiveness determined by the specific regenerative needs of the target tissue or organ^[^ ^14^ ^]^ (**Figure** **3**).

Embedding living cells within biomaterial matrices could promote proliferation, differentiation, and ECM remodeling to support tissue restoration. Scaffolds aim to mimic the natural tissue environment, where cells interact with the ECM and surrounding cells to generate functional tissue. Hydrogels, biodegradable polymers, serve as key scaffold components, improving both structural support and providing a cytocompatible environment.^[^ ^52^ ^]^ A wide range of stem cells and differentiated primary cells are integrated into these scaffolds, including mesenchymal stem cells (MSCs), pluripotent stem cells PSCs), and adipose‐derived stem cells (ADSCs).^[^ ^15^, ^53^ ^]^ The selection of the cell types depends on the intended application, being paired with a tailored scaffold design. The controlled release of growth factors and bioactive molecules could further enhance scaffold‐cell interactions, promoting angiogenesis, collagen deposition, and functional tissue remodeling.^[^ ^54^ ^]^ In addition to traditional hydrogel‐based scaffolds, bioinks for use in 3D bioprinting have been developed as a critical component for cell‐laden scaffolds.^[^ ^55^ ^]^ Bioinks are specialized formulations that encapsulate living cells, biomolecules, and ECM components, allowing precise deposition into customized tissue‐mimicking architectures.^[^ ^56^ ^]^ Through 3D bioprinting, bioinks are used to directly print stem cells such as PSCs, MSCs, and neural stem cells (NSCs), which can self‐renew and differentiate into various tissue types.^[^ ^57^ ^]^ This approach ensures high cell viability and allows for the generation of complex tissue structures that better mimic the native tissue environment. The advantage of using cell‐laden scaffolds is their capacity to provide a more physiologically relevant environment that closely mimics native tissue development.

Cell‐free (acellular) scaffolds are biomaterial‐based structures designed to support tissue regeneration by providing a biochemical and structural framework that facilitates the migration, adhesion, and proliferation of endogenous cells.^[^ ^58^ ^]^ Unlike cell‐laden scaffolds, which incorporate living cells before implantation, a key advantage of the cell‐free approach is its low immunogenicity, as no foreign cells are introduced. They are typically made from decellularized ECM, bioactive ceramics, synthetic polymers, or hydrogels.^[^ ^59^ ^]^ In contrast, cell‐laden materials demand advanced formulations, including hydrogels and composite scaffolds, that can provide a hydrated, nutrient‐rich environment while ensuring appropriate cell‐matrix interactions and functional tissue formation.^[^ ^60^ ^]^ Cell‐free scaffolds face challenges, particularly in in situ functionalization, and strategies like growth factor incorporation, nano‐topographical modifications, and bioactive coatings are developed to enhance cell recruitment and scaffold bioactivity, promoting sufficient host cell infiltration and vascularization.

The effective integration of implanted scaffolds into host tissues remains a critical challenge in tissue engineering and regenerative medicine. Whether using cell‐laden or cell‐free scaffolds, issues such as immune rejection, fibrotic encapsulation, insufficient vascularization, and poor mechanical stability can lead to scaffold failure.^[^ ^61^ ^]^ Effective biological integration requires careful consideration of biomaterial properties, immune compatibility, and the ability to support functional tissue remodeling. The next section explores how to functionalize biomaterials for transforming scaffold design to enhance integration, promote tissue remodeling, and improve long‐term therapeutic success.

## 4 Functionalization of Biomaterials and Smart Scaffolds

**Figure 4:**
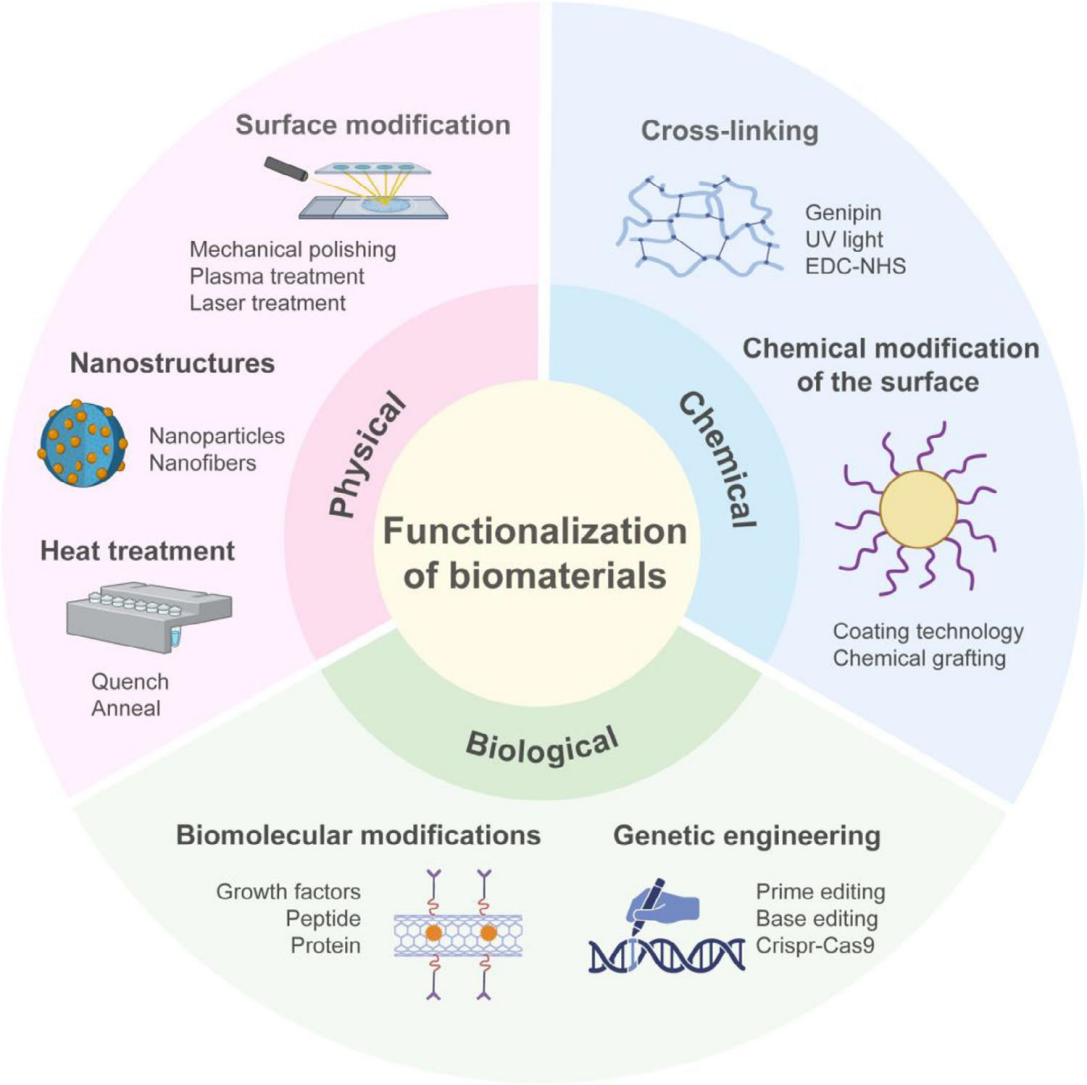
Functionalization of biomaterials by physical, chemical, and biological means. Partial graphical elements were adapted from BioRender (https://biorender.com/).

The functionalization of biomaterials has transformed the landscape of regenerative medicine by enabling scaffolds to interact dynamically with biological environments.^[^ ^62^ ^]^ Traditional biomaterials primarily focused on biocompatibility and structural support, but emerging smart scaffolds integrate bioactive properties that respond to physiological cues, enhancing tissue regeneration and integration. As shown in **Figure** **4**, physical modifications enhance surface properties and mechanical stability, promoting cell attachment and integration.^[^ ^63^ ^]^ Techniques such as plasma treatment, mechanical polishing, and electrospinning refine surface roughness, wettability, and charge distribution, enhancing protein adsorption and cellular anchorage. Nanostructure strategies, including nanoparticle integration and micro‐patterning, increase surface area‐to‐volume ratios, improving ligand presentation and cell‐matrix interactions. Additionally, engineered supramolecular hydrogels offer tunable viscoelasticity, hierarchical structuring, and controlled degradation, replicating ECM‐like dynamic properties to guide cell behavior and tissue morphogenesis.^[^ ^64^ ^]^ Chemical modifications could provide scaffolds with bioactive properties, enabling targeted interactions with cells and biomolecules. Functional groups introduced via cross‐linking strategies (e.g., EDC(1‐(3‐Dimethylaminopropyl)‐3‐ethylcarbodiimide)‐NHS(N‐Hydroxy succinimide), photopolymerization) regulate degradation rates and mechanical properties. Bioactive coatings, including peptide‐functionalized surfaces and growth factor‐conjugated materials, provide sustained bioactivity, supporting angiogenesis and host cell recruitment. Responsive materials incorporating pH‐sensitive or enzyme‐degradable linkages enable localized drug delivery, enhancing anti‐inflammatory and regenerative responses.^[^ ^65^ ^]^

Apart from biochemical responsiveness, physical design parameters such as intrinsic architecture, anisotropy, and microstructure significantly influence scaffold performance. Native tissues often exhibit complex hierarchical structures and directional mechanical properties—for instance, aligned collagen fibers in tendons or anisotropic conductivity in neural tissue.^[^ ^66^ ^]^ Advanced fabrication techniques such as directional electrospinning, gradient printing, and modular bioassembly have been applied to replicate these features.^[^ ^67^ ^]^ Anisotropic electrospun scaffolds, for example, have demonstrated superior axonal guidance in peripheral nerve regeneration.^[^ ^68^ ^]^ Similarly, gradient‐structured hydrogels can mimic osteochondral zonation, improving mechanical integration.^[^ ^69^ ^]^ The convergence of micro/nano‐structuring with bioactive patterning offers a powerful paradigm to engineer scaffolds with both functional and structural fidelity.

Advanced biofabrication techniques—such as directional electrospinning,^[^ ^70^ ^]^ gradient hydrogel printing,^[^ ^71^ ^]^ and modular organoid assembly^[^ ^69^ ^]^—are increasingly employed to achieve structural and functional biomimicry. These approaches enable precise control over scaffold anisotropy, microarchitecture, and hierarchical patterning, which are crucial for replicating oriented tissue functions (e.g., neural signal propagation, tendon alignment, osteochondral zonation). For example, aligned nanofibers guide axonal growth in nerve scaffolds, while gradient constructs support mechanically graded cartilage‐bone junctions. Such engineered anisotropy is essential for mimicking the mechanical and electrophysiological behavior of native tissues.

Traditional materials are increasingly being integrated with bioactive molecules and genetic tools to induce dynamic responsiveness. Combining cell adhesion ligands, bioactive molecules, and gene‐modulating nanoparticles can enhance cell attachment, proliferation, and differentiation, while DNA and RNA delivery systems can enable accurate gene regulation.^[^ ^72^ ^]^ Smart biomaterials further enhance functionality by dynamically responding to biological and mechanical cues.^[^ ^73^ ^]^ Mechanoresponsive polymers and magnetically responsive hydrogels adjust scaffold properties under mechanical stress, promoting tissue remodeling and enhancing mechanotransduction, which regulates cellular behavior.^[^ ^74^ ^]^ Conductive scaffolds containing carbon‐based nanomaterials support electrophysiological functions by facilitating electrical signaling in excitable tissues. Additionally, immunomodulatory biomaterials modulate inflammatory responses, reduce fibrosis, and enhance long‐term scaffold integration, ensuring better biocompatibility and functional tissue regeneration. Furthermore, we have summarized the key categories of stimuli‐responsive biomaterials, like thermo‐responsive, pH‐responsive, magnetic‐responsive, and multi‐stimuli systems, along with their mechanisms, applications, and design challenges in **Table** **1**. This provides a comparative overview to complement the narrative discussion. By combining bioactive functionalization with adaptive design, these next‐generation scaffolds not only provide structural support but also actively influence cellular behavior, offering a dynamic and clinically translatable approach to regenerative medicine.^[^ ^65^ ^]^

**Table 1:** Representative stimuli‐responsive biomaterials: classification, mechanisms, material types, biomedical applications, and design considerations.^[^ ^75^ ^]^

Material class	Stimulus type	Mechanisms of response	Material composition	Applications	Key advantages	Major limitations	Ref.
Thermo‑ responsive	Temperature change	Phase transitions; sol–gel conversion; structural reconfiguration	Poly(N‑isopropylacrylamide) (PNIPAAm)	Drug delivery; cell‑culture scaffolds	Reversible thermal response; tunability	Narrow operating temperature window	[76]
pH‑ responsive	Environmental pH	Ionisation‑induced swelling or dissolution of functional groups	Polyacrylic acid; Chitosan	Site‑specific drug release; wound healing	High biocompatibility; pH selectivity	Limited effective pH range	[77]
Photo‑ responsive	UV/visible/IR light	Photoisomerisation; photodegradation; cleavage of photo‑labile bonds	Azobenzene functionalised polymers	Optical sensors; light‑triggered drug release	Precision spatial control; remote activation	Poor light penetration in deep tissue	[78]
Electro‑ responsive	Electric field	Reversible polarisation; conductivity switching; electrochemical actuation	Polyaniline; Polypyrrole	Biosensors, neural stimulation	Fast response; easy integration with electronics	Potential cytotoxicity of conductive polymers	[79]
Magneto‑ responsive	Magnetic field	Particle alignment or motion under magnetic force	Fe₃O₄ nanoparticles; Magnetite composites	Targeted drug delivery; magnetic hyperthermia	Non‑invasive remote control	Heat generation, long‑term safety	[80]
Mechano‑ responsive	Mechanical stress/strain	Structural deformation activates mechano‑sensitive pathways	Piezoelectric ceramics; Elastomers	Tissue engineering: dynamic implants	Real‑time feedback, energy harvesting	Limited mechanical durability	[81]
Enzyme‑ responsive	Specific enzymatic activity	Enzymatic cleavage of substrate bonds or conformational change	MMP‑degradable PEG hydrogels	Targeted drug release; biosensors	High specificity; bio‑triggered degradation	Dependent on enzyme availability	[82]
Ionic‑strength responsive	Ionic concentration	Charge‑density change induces swelling or conductivity shift	Alginate; Poly(vinyl alcohol)	Drug delivery, water purification	High tunability; ion sensitivity	Performance is sensitive to ion imbalance	[83]
Multi‑stimuli responsive	Combined stimuli	Synergistic or sequential response to multiple triggers	Composite hydrogels; hybrid nanomaterials	Smart diagnostics; multifunctional devices	Versatility, tailored adaptability	Complex design and fabrication	[84]
Chemical‑ responsive	Specific chemicals	Chemical interaction induces structural or functional change	Functionalised polymers; Dendrimers	Environmental sensing; catalytic reactors	High sensitivity; signal amplification	Selectivity challenges	[85]

Recent advances in electrospinning, 3D bioprinting, and modular organoid assembly have produced constructs that better replicate native myocardium, cartilage, and neural tissue. Titanium‐based and zirconia implants are routine in dentistry, achieving ≥95% ten‐year survival despite peri‐implantitis risk.^[^ ^86^ ^]^ In orthopaedics, highly cross‐linked UHMWPE liners^[^ ^7b^ ^]^ and surface‐modified PEEK cages^[^ ^87^ ^]^ improve wear resistance and osseointegration, yet oxidative degradation and subsidence are still reported. Clinically approved collagen/β‐TCP and calcium‐phosphate cements fill non‐load‐bearing bone defects, but limited vascularisation and unpredictable resorption restrict their use in critical‐size lesions.^[^ ^88^ ^]^ Long‐term translation is further constrained by variability in host immune responses and the lack of harmonised regulatory pathways.^[^ ^89^ ^]^ Addressing these issues will require modular scaffold platforms, AI‐assisted design for cost‐effective customisation, and multicentre clinical datasets to validate performance.^[^ ^90^ ^]^

## 5 The Future of Implanted Biomaterials and Regenerative Medicine

**Figure 5:**
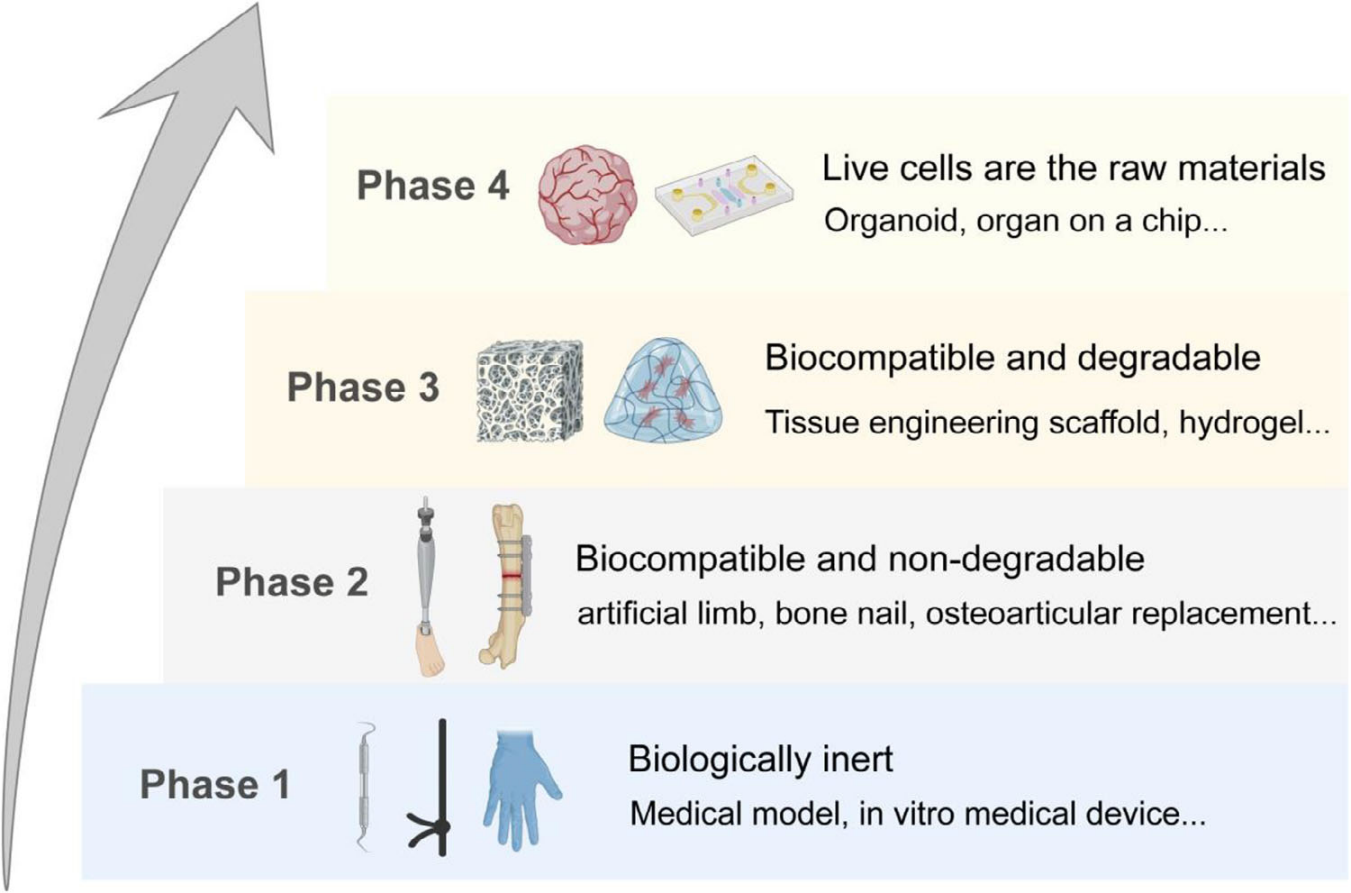
Four phases in the development of biomaterials. Phase 1: Biologically inert materials, primarily used in passive applications such as surgical tools and in vitro medical devices. Phase 2: Biocompatible yet non‐degradable materials used in long‐term implants like artificial limbs, bone nails, and joint prostheses. Phase 3: Biocompatible and degradable scaffolds (e.g., hydrogels, tissue engineering constructs), enabling temporary mechanical support and active interaction with host tissues during regeneration. Phase 4: Living cells become the primary building components, giving rise to organoids, organ‐on‐a‐chip systems, and other advanced bioengineered platforms. Partial graphical elements were adapted from BioRender (https://biorender.com/).

The future of implantable biomaterials and regenerative therapies is moving toward interactive and adaptive solutions that seamlessly integrate biological and engineering principles.^[^ ^91^ ^]^ Early biomaterials served as bioinert structural supports (Phase 1), later evolving into biocompatible but non‐degradable implants (Phase 2), such as orthopedic prosthetics. Current research focuses on biodegradable scaffolds (Phase 3), designed to guide tissue regeneration through biomaterials like hydrogels, bioactive ceramics, and engineered polymeric systems. The next transformative phase (Phase 4) envisions biointegrated materials that incorporate living cells, bioactive molecules, and gene‐editing tools to create functional tissue substitutes, organoids, and organ‐on‐a‐chip systems, enabling personalized regenerative treatments^[^ ^92^ ^]^ (**Figure** **5**). This framework reflects the shift from inert structural support to dynamic, regenerative functionality. Notable translational milestones, for example, the first FDA‐approved resorbable coronary scaffold (2016) and the clinical implementation of 3D‐bioprinted tissues, underscore the stepwise progression between these phases.

Emerging technologies, including 3D bioprinting, nanotechnology, and advanced biofabrication enable the precise design of scaffolds that mimic native tissue architecture and function. Smart biomaterials, including shape‐memory polymers, magneto‐responsive hydrogels, and electrically conductive scaffolds, are paving the way for functional tissue restoration by responding to mechanical, chemical, and electrical cues within the physiological environment.^[^ ^93^ ^]^ Furthermore, genome‐integrated biomaterials and organoid engineering are opening new avenues for tissue repair and organ replacement, utilizing patient‐specific cellular and genetic information for highly personalized regenerative therapies.^[^ ^94^ ^]^

However, major challenges remain in clinical translation, including regulatory complexities, scalability of manufacturing, and long‐term biocompatibility.^[^ ^95^ ^]^ Standardization of production techniques and biomaterial characterization is crucial for large‐scale production, while ethical considerations regarding genetically modified scaffolds and bioengineered tissues must be addressed. Moving forward, interdisciplinary collaboration among material scientists, bioengineers, and clinicians will be critical in bridging the gap between cutting‐edge biomaterial research and real‐world medical applications. With continued progress in bioengineering, in situ tissue regeneration, and precision medicine, next‐generation biomaterials hold the potential to revolutionize regenerative medicine, bringing functional tissue replacement and organ regeneration closer to widespread clinical application. While patient‐specific biomaterials offer improved compatibility, their clinical translation is constrained by high fabrication costs, long production times, and regulatory complexity. In contrast, standardized materials are scalable but often lack customization. Emerging solutions, including modular scaffold designs and semi‐customized bioprinting, may help balance personalization with feasibility.^[^ ^96^ ^]^

Looking forward, the evolution of bioinspired implants is expected to follow a phased trajectory. In the near term, research will focus on standardizing bioinks and refining modular assembly processes to improve manufacturing reproducibility and scalability. Computational modeling and machine learning are progressively applied to optimize scaffold architecture, predict host response, and design smart materials with programmed degradation and stimuli responsiveness. For instance, AI‐based algorithms can assist in aligning mechanical gradients of implants with target tissues, guiding the design of anisotropic or hierarchical scaffolds for patient‐specific conditions. In the long term, convergence with bioelectronics and tissue‐on‐chip technologies may enable biohybrid implants capable of closed‐loop therapeutic modulation and real‐time physiological monitoring. These developments are expected to bridge the translational gap between engineering innovation and clinical implementation, driving next‐generation regenerative therapies.

Early studies suggest that machine‐learning techniques can help analyse existing datasets on scaffold composition, degradation rate and mechanical properties, offering preliminary guidance for material selection and parameter screening. Although fully predictive modelling of complex cell–material interactions is not yet feasible, data‐driven approaches are beginning to identify trends that inform porosity or composition choices for specific tissues. In parallel, proof‐of‐concept “inverse design” algorithms are being explored to propose candidate scaffold geometries that meet multiple design constraints.^[^ ^97^ ^]^ Continued progress will depend on larger, well‐curated datasets, integration with physics‐based models and rigorous experimental validation. As such tools mature, they are expected to complement—rather than replace—traditional experimental workflows in the rational design of next‐generation biomaterials.^[^ ^98^ ^]^

## 6 Conclusion: Bridging the Gap Between Technology and Biology

The interdisciplinary integration of technology and biology in implantable biomaterials is transforming regenerative medicine, or methodologically, developing appropriate methods to enhance tissue regeneration and functional integration by combining passive responses with active design and regulation. Traditional materials primarily relied on structural support, reacting passively to biological environments. Advanced technologies now allow biomaterials to sense and respond to physiological cues, dynamically adjusting properties to promote cellular activity, immune modulation, and controlled degradation. The synergy of passive compatibility and active bioengineering has given rise to the next generation of scaffolds that replicate natural tissues and enhance repair functions through biochemical signals and mechanical adaptation. Overcoming challenges in clinical translation, large‐scale production, and regulatory approval remains critical for final patient application. Future biomaterials will bridge technology and biology by combining responsive design with biological control, enabling self‐regulating tissue architectures and unlocking personalized and regenerative medical solutions. Despite progress in material design, clinical translation is still limited due to key challenges. Animal models often fail to predict human responses because of species‐specific immune differences and simplified disease conditions. This limits the reliability of preclinical data, especially for long‐term safety and integration. Addressing these gaps requires more representative models and improved immunomodulatory strategies.^[^ ^99^ ^]^

For such advances to be developed in a clinical environment, collaborations between scientists and clinicians must be established. Input from regulators, ethicists, and health economic experts is critical to success. We must also throw of the shackles that constrain our thinking about manufacturing. These advanced materials and structures certainly demand next‐level manufacturing. Such manufactory lines will be multidimensional and will require the use of AI to get optimal results. These new manufacturing approaches also require us to think innovatively when it comes to “in‐line” monitoring and characterization. As biomaterials become more biologically active, ethical and safety concerns grow accordingly. In advanced Phase 4 systems such as organoids, genetic modification raises potential risks of off‐target effects and heritable changes, necessitating strict oversight and ethical scrutiny. Likewise, nanomaterials face unresolved challenges regarding long‐term toxicity, accumulation, and in vivo degradation. Addressing these issues will require coordinated interdisciplinary regulation, long‐term assessment, and international consensus.

## Conflict of Interest

The authors declare no conflict of interest.

## Author Contributions

Q.G. did conceptualization, investigation, wrote the original draft, review & editing. R.Y. and D.S. did conceptualization, investigation, wrote the original draft, review & editing. J.C. did conceptualization, funding acquisition, supervision, wrote the original draft, review & editing. G.W. did conceptualization, funding acquisition, supervision, wrote the original draft, review & editing.

## Data Availability

Data supporting the plots used in this study are available from the corresponding author upon request.
